# Case report: Transected Hickman catheter and its thrombotic occlusion in a patient with idiopathic pulmonary arterial hypertension—can a catheter replacement be avoided?

**DOI:** 10.3389/fcvm.2023.1230417

**Published:** 2023-07-20

**Authors:** Grzegorz Sławiński, Piotr Zieleniewicz, Anna Faran, Alicja Dąbrowska-Kugacka, Marcin Kurzyna, Maciej Kempa, Ludmiła Daniłowicz-Szymanowicz, Ewa Lewicka

**Affiliations:** ^1^ Department of Cardiology and Electrotherapy, Faculty of Medicine, Medical University of Gdańsk, Gdańsk, Poland; ^2^ Club 30, Polish Cardiac Society, Warsaw, Poland; ^3^ Department of Pulmonary Circulation, Thromboembolic Diseases and Cardiology, Centre of Postgraduate Medical Education in EHC Otwock, ERN-Lung Member, Otwock, Poland

**Keywords:** hickman and broviac catheters, epoprostenol, pulmonary hypertension, thrombosis, repair kit, pulmonary hypertension

## Abstract

A 25-year-old female with idiopathic pulmonary arterial hypertension (PAH), who had a Hickman catheter implanted for continuous intravenous epoprostenol infusion, was admitted to the clinic after inadvertently cutting the catheter with nail scissors during a routine dressing change. Approximately 7 cm of the external segment of the Hickman catheter remained intact, with the distal end knotted by paramedics. A decision was made to repair the damaged Hickman catheter. However, it was discovered that its lumen was completely occluded by thrombosis. Therefore, catheter patency was mechanically restored using a 0.035-inch stiff guidewire in a sterile operating theatre setting, under fluoroscopy guidance. Successful aspiration and catheter flushing were achieved. Continuity of the Hickman catheter was then restored using a repair kit (Bard Access Systems) as per the manufacturer's instructions, with no visible leakage thereafter. Epoprostenol infusion through the Hickman catheter was resumed 24 h later, and the patient was discharged in good general condition two days afterward.

## Introduction

Epoprostenol is a drug that significantly reduces pressure in the pulmonary circulation, requiring continuous intravenous infusion, typically through tunneled catheters. In situations where the continuous supply of epoprostenol is disrupted, infusion should be restored as soon as possible, even via a peripheral vein, until central access is obtained. In our patient, the significant challenge was not only restoring the continuity of the catheter but also addressing a thrombotic obstruction at the point of intersection. We present a procedure that can be used in these patients, postponing the need for catheter reimplantation. Moreover, tunneled central venous catheters provide long-term intravenous access for parenteral nutrition, fluid resuscitation, antibiotics, chemotherapy, and hemodialysis. Therefore, the management strategy for such complications, as described in our case report, may be applicable to a large group of patients (1).

## Case description

**Figure 1:**
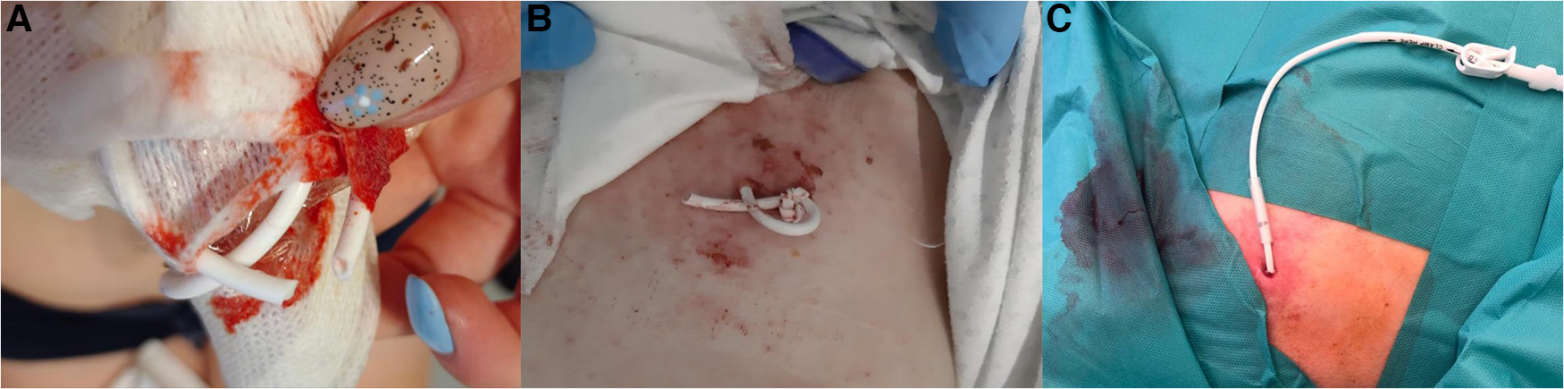
(Panel **A**) photograph provided by the patient after inadvertently cutting the catheter. (Panel **B**) Photograph showing the catheter tied in a knot by paramedics. (Panel **C**) Photograph taken immediately after the procedure to restore the continuity of the Hickman catheter.

A 25-year-old female with idiopathic pulmonary arterial hypertension (PAH), who had a Hickman catheter implanted for continuous intravenous epoprostenol infusion, was admitted to the clinic after inadvertently cutting the catheter with nail scissors during a routine dressing change (Figure 1A).

The patient had been receiving treatment with epoprostenol (100 ng/kg/min), macitentan, and sildenafil for 3 years, with excellent outcomes (NYHA class I). Upon admission, due to the damaged Hickman catheter and resulting abrupt discontinuation of epoprostenol, she was in a severe condition, with dyspnea at rest, low blood oxygen saturation (72%) and hypotension (83/55 mmHg). A central catheter for epoprostenol infusion was immediately placed and the patient's condition gradually improved.

**Figure 2:**
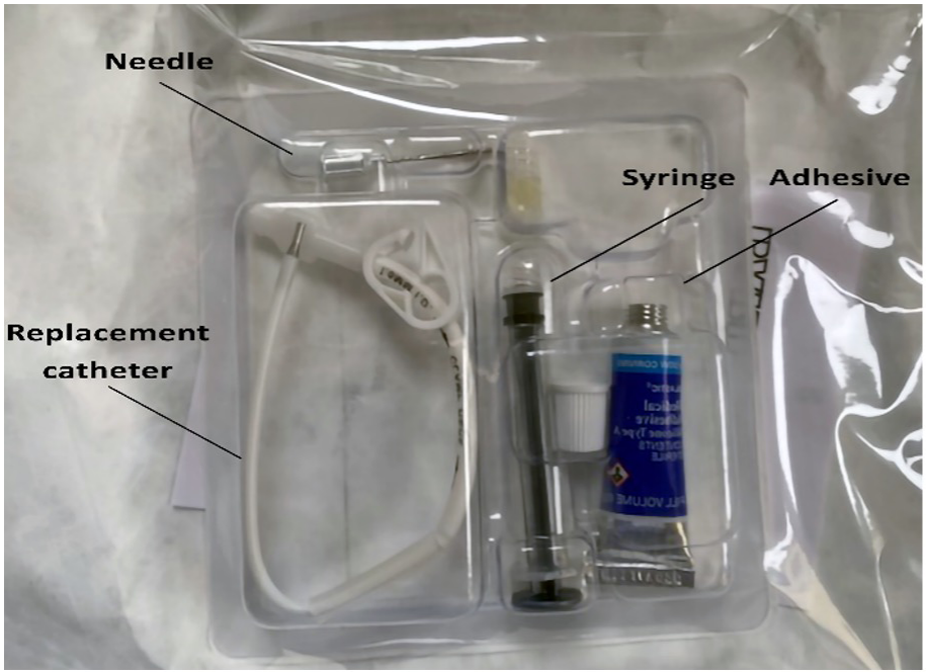
Repair kit contents by Bard Access Systems, Inc. (own material).

Approximately 7 cm of the external segment of the Hickman catheter remained intact, with the distal end knotted by paramedics (Figure 1B). The case was discussed with a leading pulmonary hypertension center, and a decision to repair the damage to the Hickman catheter was made. However, it was discovered that its lumen was completely occluded by thrombosis. Therefore, the catheter patency was mechanically restored using a 0.035-inch stiff guidewire in sterile operating theatre setting, under fluoroscopy guidance. Successful aspiration and catheter flushing were achieved. The continuity of the Hickman catheter was then restored using a repair kit (Bard Access Systems) as per the manufacturer's instructions (Figure 1C). First, the syringe barrel was filled with adhesive and connected to a blunt needle. The remaining external segment of the catheter was cut at a 90-degree angle, proximal to the damaged area. Then, the existing catheter tip was connected with the replacement catheter using a stent, leaving a 3 mm gap between them. The adhesive from the previously prepared syringe was applied to this gap, and the catheter ends were brought closer together. The adhesive was applied externally around the spliced joint of the catheter, covering a length of approximately 2.5 cm. The sleeve was slid to the joint, and the space within the sleeve was filled with adhesive from both sides (see Figure 2). The catheter was rinsed with heparinized saline, and the repaired area was immobilized with a splint and then secured with a sterile dressing. There was no visible leakage following the procedure. In the perioperative period and for the next 3 days, cefazolin was administered to prevent infectious complications. Epoprostenol infusion through a Hickman catheter was resumed 24 h later, and the patient was discharged in good general condition two days afterward. Currently, after 65 days of follow-up, the patient remains cardiovascularly stable and in a good condition with a properly functioning repaired Hickman catheter.

## Discussion

According to the ESC 2022 guidelines on pulmonary hypertension, parenteral prostacyclin analogues, e.g., epoprostenol, are recommended in patients with high-risk PAH, and such treatment was used in our patient with a very good clinical effect (2). Epoprostenol is administered using a Hickman catheter, which is a tunneled central venous access line, with thrombosis and infections being the most common complications. Catheter breakage is also a possible complication (3). The use of a repair kit is an inexpensive and effective solution in the event of damage to the external catheter segment, which allows its continued use with good long-term effect without an increased risk of infection (4). Hwang et al. reported a mean dwell time of 79 days after the repair of a catheter, a period similar to our follow-up time, with no significant difference in the overall survival time of the repaired catheters compared to undamaged catheters (143.4 vs 145.4 days, *p* = 0.79) (5). Newer data from Wouters et al. shows even better results—on average, the repair prolonged the catheter's survival time by 828 days (6). However, it's not always possible to repair a damaged catheter. In the pediatric patient population, fractures of the external component of the Hickman line occurred in 11 out of 91 patients (12%); only 3 of these fractures were suitable for repair, while the remaining 8 required removal and re-insertion (7).

In our patient, thrombotic obstruction of the catheter at the point of its intersection was a significant difficulty. This prevented the use of alteplase, which, when administered locally in the case of catheter thrombosis, effectively restores its patency (8). Therefore, the decision was made to attempt to cross the thrombosis with a 0.035-inch guidewire. Intraluminal thrombosis, either partial or complete, accounts for 25% of all cases of obstruction of tunneled IV catheters. In addition to alteplase, some centers also develop protocols with a 6-hour administration of urokinase (9). For the pediatric population, however, Zhang et al. followed a protocol involving first an attempt with a high-pressure flush using diluted heparinized saline (10 units per ml) in a 2 ml syringe followed by an attempt to flush the line with tissue plasminogen activator (0.5 mg in 2 ml for patients <10 kg and 2 mg in 2 ml for patients >10 kg) (7). In the event of failure of pharmacological treatment (or inability to use it—as in our case due to thrombosis reaching the very end of the damaged catheter), a guidewire or a snare can be used to remove the clot from the catheter (10). This makes it possible to repair the catheter and avoid its replacement.

Lastly, it should be underlined that in situations such as in the presented case report, epoprostenol infusion should be restored as soon as possible, even via a peripheral vein, until central access is obtained.

## Patient perspective

The implantation of a tunneled catheter for the infusion of epoprostenol may lead to complications and discomfort for the patient. From a patient's perspective, the ability to repair the existing catheter provides a valuable opportunity to continue the treatment with epoprostenol, eliminating the need for catheter reimplantation.

## Data Availability

The original contributions presented in the study are included in the article, further inquiries can be directed to the corresponding author.
